# Hypersusceptibility mechanism of Tenofovir-resistant HIV to EFdA

**DOI:** 10.1186/1742-4690-10-65

**Published:** 2013-06-24

**Authors:** Eleftherios Michailidis, Emily M Ryan, Atsuko Hachiya, Karen A Kirby, Bruno Marchand, Maxwell D Leslie, Andrew D Huber, Yee T Ong, Jacob C Jackson, Kamalendra Singh, Eiichi N Kodama, Hiroaki Mitsuya, Michael A Parniak, Stefan G Sarafianos

**Affiliations:** 1 Christopher Bond Life Sciences Center, Department of Molecular Microbiology & Immunology, University of Missouri, Columbia, MO 65211, USA; 2 Clinical Research Center, Department of Infectious Diseases and Immunology, National Hospital Organization Nagoya Medical Center, Nagoya 4600001, Japan; 3 Division of Emerging Infectious Diseases, Tohoku University, Sendai 980-8575, Japan; 4 Department of Internal Medicine, Kumamoto University, Kumamoto 860-8556, Japan; 5 Experimental Retrovirology Section, HIV/AIDS Malignancy Branch, NIH, Bethesda, MD 20892, USA; 6 Department of Microbiology and Molecular Genetics, University of Pittsburgh, Pittsburgh, PA 15219, USA; 7 Department of Biochemistry, University of Missouri, Columbia, MO 65211, USA

**Keywords:** HIV-1, RT, EFdA, K65R

## Abstract

**Background:**

The K65R substitution in human immunodeficiency virus type 1 (HIV-1) reverse transcriptase (RT) is the major resistance mutation selected in patients treated with first-line antiretroviral tenofovir disoproxil fumarate (TDF). 4'-ethynyl-2-fluoro-2'-deoxyadenosine (EFdA), is the most potent nucleoside analog RT inhibitor (NRTI) that unlike all approved NRTIs retains a 3'-hydroxyl group and has remarkable potency against wild-type (WT) and drug-resistant HIVs. EFdA acts primarily as a chain terminator by blocking translocation following its incorporation into the nascent DNA chain. EFdA is in preclinical development and its effect on clinically relevant drug resistant HIV strains is critically important for the design of optimal regimens prior to initiation of clinical trials.

**Results:**

Here we report that the K65R RT mutation causes hypersusceptibility to EFdA. Specifically, in single replication cycle experiments we found that EFdA blocks WT HIV ten times more efficiently than TDF. Under the same conditions K65R HIV was inhibited over 70 times more efficiently by EFdA than TDF. We determined the molecular mechanism of this hypersensitivity using enzymatic studies with WT and K65R RT. This substitution causes minor changes in the efficiency of EFdA incorporation with respect to the natural dATP substrate and also in the efficiency of RT translocation following incorporation of the inhibitor into the nascent DNA. However, a significant decrease in the excision efficiency of EFdA-MP from the 3’ primer terminus appears to be the primary cause of increased susceptibility to the inhibitor. Notably, the effects of the mutation are DNA-sequence dependent.

**Conclusion:**

We have elucidated the mechanism of K65R HIV hypersusceptibility to EFdA. Our findings highlight the potential of EFdA to improve combination strategies against TDF-resistant HIV-1 strains.

## Background

Human immunodeficiency virus type 1 (HIV-1) reverse transcriptase (RT) is the major target of antiretroviral drug treatments. RT inhibitors constitute the largest class of HIV-1 drugs and are grouped in two separate categories. The first category consists of the nucleos(t)ide RT inhibitors (NRTIs), which are analogs of the natural nucleosides. Most NRTIs lack a 3’-OH and act as chain terminators by blocking DNA polymerization [1-8]. The other group includes the nonnucleoside RT inhibitors (NNRTIs), which are non-competitive RT inhibitors with respect to either dNTP or nucleic acid substrates and block DNA synthesis by binding to a hydrophobic pocket of RT [9-15]. Highly Active Antiretroviral Therapies (HAART) are based on combinations of antiretrovirals and have helped extend the lives of HIV-1 patients. However, the efficacy of combination therapies is being challenged by the selection of drug-resistant variants of HIV-1.

There are two major mechanisms of NRTI resistance [16,17]. The first is the discrimination mechanism, which is based on decreased incorporation of the nucleotide analog into the elongating DNA over the canonical dNTP substrate [16,18-21]. An example of this type of resistance is conferred by the M184V mutation, which decreases HIV susceptibility to lamivudine (3TC) and emtricitabine (FTC) [20-24]. The second mechanism is the excision mechanism, which is based on the enhanced ability of the mutant RT to remove the chain-terminating inhibitor from the DNA terminus [25-28] through a phosphorolytic reaction that uses primarily adenosine triphosphate (ATP) as a substrate. Upon removal of the inhibitor DNA synthesis resumes. The excision reaction is facilitated by Excision Enhancement Mutations (EEMs), typically M41L, D67N, K70R, T215Y/F, L210W, and K219E/Q, which are also known as Thymidine Associated Mutations (TAMs) because they were historically linked to resistance to thymidine analogs AZT and d4T [29,30].

Tenofovir disoproxil fumarate (TDF) is one of the most prescribed anti-HIV drugs, and is described as a key component of all first-line regimens in the DHHS HIV guidelines (http://aidsinfo.nih.gov/contentfiles/lvguidelines/adultandadolescentgl.pdf). The K65R mutation in HIV-1 RT is the signature mutation selected during tenofovir-based therapy. Viruses carrying K65R have reduced susceptibility to tenofovir and other NRTIs, but remain susceptible to zidovudine (AZT) [31-36]. This mutation has also been associated with a reduction in viral replication capacity, NRTI excision, NRTI incorporation, and dNTP incorporation [37-43]. Recent crystallographic data suggest that the K65R mutation disrupts the interaction between the side chains of 65R and 72R resulting in structural changes that lead to NRTI resistance [44].

We have previously shown that a series of NRTIs with 4’-substitutions and a 3’-OH group are very potent inhibitors of WT and multi-drug resistant HIV-1. The most effective of these compounds is the adenosine analog 4’-ethynyl-2-fluoro-2’-deoxyadenosine (EFdA) [45,46]. We have demonstrated that EFdA acts in a DNA-sequence specific manner, primarily inhibiting DNA synthesis as an immediate chain terminator, but less often, at some DNA sequences can also act as a delayed chain terminator [46]. Compounds that exhibit this novel mechanism of inhibition have been dubbed Translocation Defective Reverse Transcriptase Inhibitors (TDRTIs) [46].

In an effort to investigate the effect of EFdA against drug-resistant strains of HIV-1 we found that RT mutation K65R confers hypersusceptibility to EFdA. We carried out a series of biochemical experiments to elucidate the mechanism of this phenomenon and we propose here that K65R increases the susceptibility to EFdA mainly by suppressing the ATP- or PPi-dependent repair of EFdA-MP-terminated DNA. Understanding the molecular basis of K65R hypersusceptibility to EFdA may lead to new and more effective combination therapies.

## Results

### The K65R RT mutation enhances susceptibility of HIV to EFdA

In order to determine the susceptibility of HIV-1 to EFdA we performed single infectivity viral replication assays according to the experimental procedures described in Methods section. We used as a positive control of resistance to K65R HIV-1 the nucleotide analog TDF. Table 1 shows that K65R-containing viruses are at least 2.5-fold more susceptible to EFdA than WT viruses. In contrast, there was a 3-fold resistance to tenofovir caused by K65R RT mutation.

**Table 1:** **EC**_**50 **_**determination of EFdA and TDF in single cycle cell-based assays**

**Virus**	**EC**_**50**_ **± SD (nM) (Fold change)**	
	**EFdA**	**TDF**
WT	3.2 ± 0.7	32 ± 6
	(1)	(1)
K65R	1.3 ± 0.4	96 ± 3
	(0.4)	(3)

The data show “mean value ± standard deviation” obtained from the results of at least three independent experiments (P < 0.013). The relative increase in EC_50_ values against K65R virus compared with WT virus is given in parentheses.

### The K65R mutation enhances susceptibility of RT to EFdA-TP

**Figure 1:**
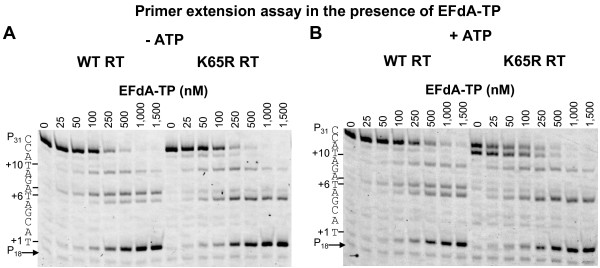
**Inhibition of WT and K65R RT-catalyzed DNA synthesis by EFdA-TP.** T_d31_/P_d18-P0_ was incubated with WT or K65R HIV-1 RT for 50 minutes in the presence of 1 μM dNTPs, MgCl_2_ and increasing concentrations of EFdA-TP (0–1,500 nM). The experiment was carried out in the (**A**) absence or (**B**) presence of 3.5 mM ATP. The template sequence is shown next to the gels and the numbers indicate the points of EFdA-TP incorporation (+1, +6 and +10).

In order to recapitulate the HIV hypersusceptibility to EFdA observed in cell-based assays and determine the biochemical mechanism of this phenomenon we carried out a series of biochemical experiments. We used a primer extension assay to compare the effect of EFdA-TP on DNA-dependent DNA polymerization by WT and K65R RTs. In order to assess the effect of ATP-based excision on the susceptibility of RT to EFdA-TP we performed the reactions in the absence (Figure 1A) and in the presence of ATP (Figure 1B). In the absence of ATP, any changes in the susceptibility to the inhibitor would be caused by the “decreased incorporation” mechanism as there are no NRTI excision events under these conditions. However, changes in inhibitor susceptibility in the presence of ATP could be caused either by “changes in inhibitor incorporation” or “changes in inhibitor excision”, or both. Figure 1 and Table 2 show that RT mutation K65R causes hypersusceptibility to EFdA-TP. In the presence of ATP the K65R mutation caused a 2.5-fold increase in susceptibility to EFdA-TP (Table 2). These data are consistent with the results from the cell-based assays shown in Table 1. The ~2-fold effect of ATP-based excision on the hypersusceptibility to EFdA suggests that excision is the major mechanism of this phenomenon and is further characterized in the subsequent experiments.

**Table 2:** Enhancement of hypersusceptibility to EFdA-TP under ATP-based excision conditions

**Enzyme**	**IC**_**50**_**(nM) of EFdA-TP ± SD (Fold change)**		**Hypersusceptibility enhancement in the presence of ATP**
	**Without ATP**	**With 3.5 mM ATP**	
WT RT	186 ± 40^a^ (1)^b^	318 ± 99^a^ (1)^b^	1^c^
K65R RT	125 ± 28^a^ (0.7)^b^	131 ± 28^a^ (0.4)^b^	1.8^c^

^a^The data show “mean value ± standard deviation” obtained from the results of at least four independent experiments (P < 0.011).

^b^The relative increase in IC_50_ value in K65R RT compared with WT RT without, or with ATP is given in parentheses.

^c^The effect of ATP-based excision on hypersusceptibility is calculated by the relative change in IC_50_ for K65R compared to WT RT without ATP/the relative change in IC_50_ for K65R compared to WT RT with ATP.

The T_d31_/P_d18-P0_ DNA substrate (Table 3) allows incorporation of dATP analogs opposite template dTs at positions “1”, “6” and “10” (Figure 1). The stopping pattern of the gels showed that EFdA-TP caused major pauses at all possible points of incorporation (positions 1, 6 and 10), suggesting that EFdA-TP inhibits RT mainly as an immediate chain terminator at the point of incorporation. Interestingly, EFdA-TP caused an additional strong stop of WT RT at position “+7”, which is one nucleotide after its incorporation (Figure 1), thereby acting as delayed chain terminator at this site. Hence, this appears to be a sequence-dependent phenomenon, as we did not observe delayed chain extension at positions +2 and +11 and there was no pause at this site in the absence of inhibitor (Figure 1).

**Table 3:** Sequences of oligonucleotides used in this study

**Polymerization assays**	
T_d31_	5’CCA TAG ATA GCA TTG GTG CTC GAA CAG TGA C
P_d18-P0_	5’Cy3 GTC ACT GTT CGA GCA CCA
T_d26_	5’CCA TAG ATA GCA TTG GTG CTC GAA CA
P_d18-P5_	5’Cy3 TGT TCG AGC ACC AAT GCT
T_d31A_	5’AAA AAA AAA TGG ATA CAT ATG GTT AAA GTA T
P_d21_	5’Cy3 ATA CTT TAA CCA TAT GTA TCC
**Footprinting assays**	
T_d43_	5’Cy3 CCA TAG ATA GCA T TG GTG CTC GAA CAG TGA CAA TCA GTG TAG A
P_d30_	5’TCT ACA CTG ATT GTC ACT GTT CGA GCA CCA

### The K65R RT mutation does not enhance susceptibility to EFdA by significantly affecting incorporation of the inhibitor

To determine the biochemical mechanism of increased K65R HIV inhibition by EFdA we examined several possible mechanisms. The first hypothesis was that the K65R RT mutation selectively enhances incorporation of the EFdA-TP inhibitor into DNA because of changes in kinetic parameters such as binding or turnover rate of inhibitor incorporation. To evaluate this hypothesis we performed single nucleotide incorporation assays under steady state conditions. In order to eliminate a sequence-dependent bias we used three different template/primers (T/P) (T_d26_/P_d18-P5_, T_d31_/P_d18-P0_, and T_d31A_/P_d21_ [Table 3]). Our results showed that under these conditions the ratio of the incorporation efficiency (*k*_cat_/K_m_) of EFdA-TP and the incorporation efficiency of dATP by K65R RT was between 0.8 and 1. These results suggest that the K65R mutation does not have a significant effect on the binding and incorporation of EFdA-TP (Table 4).

**Table 4:** Steady state kinetic parameters for EFdA-TP and dATP incorporation by WT and K65R HIV-1 RTs

**Enzyme**	**dNTP**	***K***_***m***_**(μM)**	***k***_**cat**_**(min**^**-1**^**)**	***k***_**cat**_**/*K***_***m***_**(min**^**-1**^** · μM**^**-1**^**)**	**Selectivity**^**a**^	**Fold change**^**b**^
**T**_**d26**_**/P**_**d18-P5**_						
WT RT	dATP	2.38 ± 0.27	1.58 ± 0.12	0.66	1	1
	EFdA-TP	0.66 ± 0.04	3.26 ± 0.42	4.94	7.5	
K65R RT	dATP	5.99 ± 0.81	0.92 ± 0.14	0.15	1	0.8
	EFdA-TP	1.83 ± 0.34	1.74 ± 0.42	0.95	6.3	
**T**_**d31**_**/P**_**d18-P0**_						
WT RT	dATP	0.33 ± 0.07	6.32 ± 0.14	19.15	1	1
	EFdA-TP	0.23 ± 0.01	4.73 ± 0.33	20.57	1.1	
K65R RT	dATP	0.42 ± 0.01	3.62 ± 0.71	8.62	1	1
	EFdA-TP	0.31 ± 0.02	3.07 ± 0.15	9.90	1.1	
**T**_**d31A**_**/P**_**d21**_						
WT RT	dATP	0.37 ± 0.08	3.64 ± 0.57	9.84	1	1
	EFdA-TP	0.19 ± 0.06	3.54 ± 0.44	18.63	1.9	
K65R RT	dATP	1.06 ± 0.11	3.68 ± 0.28	3.47	1	0.9
	EFdA-TP	0.53 ± 0.05	3.38 ± 0.29	6.38	1.8	

Values are mean ± S.D. of two to four independent experiments and were determined from Michaelis-Menten equation using GraphPad Prism 4.

^a^Selectivity is the ratio of the incorporation efficiency (*k*_cat_*/K*_*m*_) of EFdA-TP over that of dATP ([*k*_cat_*/K*_*m*_]_EFdA-TP_ / [*k*_cat_*/K*_*m*_]_dATP_).

^b^Fold Change is the ratio of the selectivity in K65R over the selectivity in WT RT.

### The K65R RT mutation does not enhance susceptibility to EFdA by significantly affecting enzyme translocation on EFdA-MP-terminated template/primers

**Figure 2:**
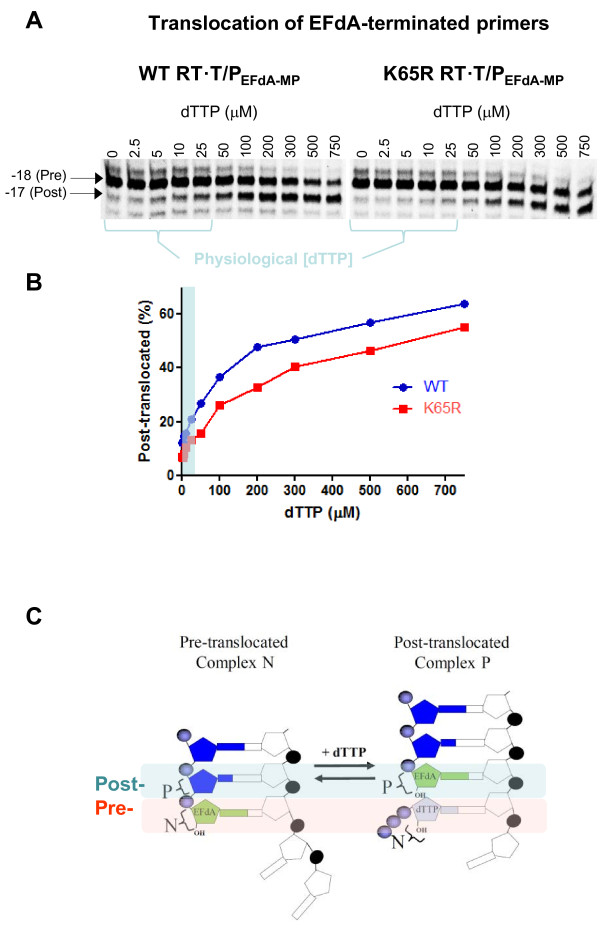
**Effect of K65R mutation on the translocation state of RT bound to T/P**_**EFdA-MP**_**.** (**A**) The translocation state of HIV-1 RT after EFdA-MP incorporation was determined using site-specific Fe^2+^ footprinting. T_d43_/P_d30-EFdA-MP_ (100 nM) with 5'-Cy3-label on the DNA template was incubated with WT or K65R HIV-1 RT (600 nM) and various concentrations of the next incoming nucleotide (dTTP). The complexes were treated for 5 minutes with ammonium iron sulphate (1 mM) and resolved on a polyacrylamide 7 M urea gel. An excision at position −18 indicates a pre-translocation complex, while the one at position −17 represents a post-translocation complex. (**B**) The post-translocated complexes were determined from the gels and plotted using GraphPad Prism. Light blue indicates the physiological dNTP concentrations. (**C**) Schematic representation of the position of EFdA-MP-terminated primers at the pre- and post-translocated sites.

We have previously shown that the inability of RT to form a stable ternary complex with T/P_EFdA-MP_ and the next complementary dNTP was due to the inability of the 3’-terminal EFdA-MP primer to efficiently translocate from the nucleotide binding site (N site, which is also the pre-translocation site) to the post-translocation primer site (P site or the post-translocation site) [46]. Hence, another possible mechanism by which the K65R mutation could enhance susceptibility to EFdA is by further suppressing translocation of RT on the EFdA-MP-terminated T/P. To evaluate this hypothesis we used the site-specific Fe^2+^ foot-printing assay [46,47] to assess the translocation state of WT and K65R RT·T/P_EFdA-MP_ complexes in the absence, and in the presence of varying concentrations of the next incoming dNTP. Figure 2 shows that EFdA-TP blocked translocation and acted as a strong TDRTI against both WT and K65R RTs. At physiological dNTP concentrations (1-25 μM) we observed a 1.5-fold decrease in the translocation efficiency of K65R compared to WT RT under these conditions. To determine whether the lower amount of observed translocated K65R RT·T/P_EFdA-MP_ complex (Figure 2) is due to a decreased affinity of K65R RT for EFdA-MP-terminated T/P we studied the effect of K65R on the formation of RT·T/P_EFdA-MP_ binary complex using gel-shift assays. Data in Additional file 1: Figure S1 show that WT RT binds EFdA-MP-terminated T/P only slightly stronger than K65R RT (~1.3-fold). Therefore, the small differences in the selectivity, translocation activity, and DNA binding of WT and K65R RTs were not sufficient to explain the hypersusceptibility we observed in cell-based and RT assays with EFdA and EFdA-TP respectively. Therefore, in the following experiments we examined whether the K65R substitution could enhance susceptibility to EFdA by suppressing the ability of RT to unblock EFdA-MP-terminated primers.

### ATP- and PPi-dependent Excision/Rescue of EFdA-MP

**Figure 3:**
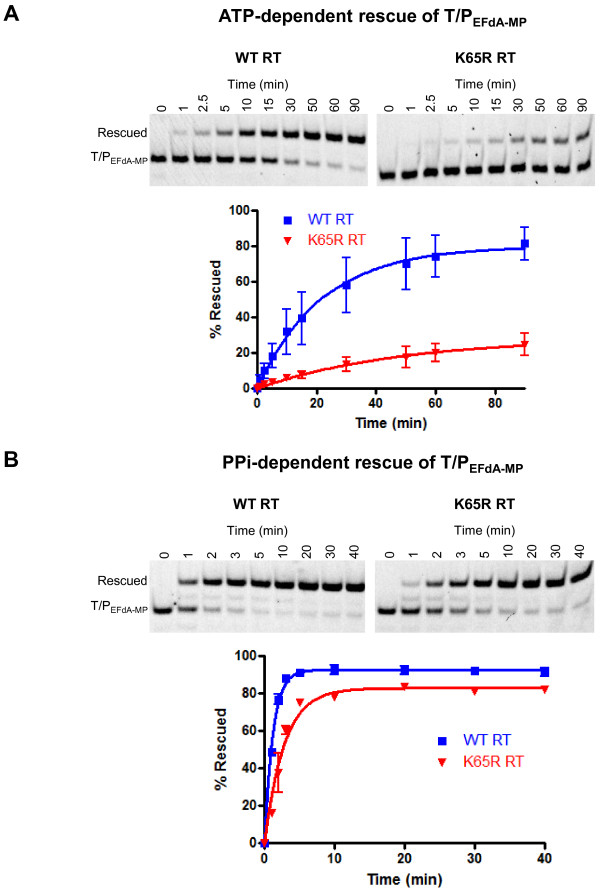
**ATP- and PPi-dependent rescue of EFdA-MP terminated primers by WT and K65R RTs.** (**A**) ATP-dependent rescue of T_d31_/P_d18-P0-EFdA-MP_. Purified T_d31_/P_d18-P0-EFdA-MP_ was incubated with WT or K65R RT in the presence of 10 mM MgCl_2_, 3.5 mM ATP, 100 μM dATP, 0.5 μM dTTP, and 10 μM ddGTP at 37°C. Aliquots of the reaction were stopped at the indicated time points (0–90 min). The results of at four independent experiments were plotted using one site hyperbola in Graphpad Prism 4. (**B**) PPi-dependent rescue of T_d31_/P_d18-P0-EFdA-MP_. Purified T_d31_/P_d18-P0-EFdA-MP_ was incubated with WT or K65R RT in the presence of 6 mM MgCl_2_, 150 μM PPi, 100 μM dATP, 0.5 μM dTTP, and 10 μM ddGTP at 37°C. Aliquots of the reaction were stopped at the indicated time points (0–40 min). The results of two independent experiments were plotted using one site hyperbola in Graphpad Prism 4.

We have previously demonstrated that using simple pyrophosphorolysis reactions (in the absence of concurrent DNA polymerization) is not an effective way to monitor unblocking of EFdA-MP-terminated primers. This is because the net phosphorolysis is limited from the apparently facile reincorporation of the newly excised EFdA-TP [46]. Hence, to better study the potential role of the excision mechanism in EFdA resistance we employed rescue assays, where in addition to the ATP or PPi which are used as unblocking reagents, we also include dNTPs that compete with and prevent reincorporation of EFdA-TP, and also allow further DNA synthesis. For these experiments we used as a substrate nucleic acid having at the 3’-primer terminus EFdA-MP (T_d31/_P_d18-P0-EFdA-MP_). Using ATP as the pyrophosphate donor, we found that the initial rates of the rescue reactions were 2.8-fold slower by K65R than by WT RT (Figure 3A). A 6.5-fold decrease was also observed in PPi-based rescue (Figure 3B). As previously reported [48] the PPi-rescue was faster than the ATP-based rescue assay. Whereas the PPi-based hydrolysis is exactly the opposite of DNA synthesis in reverse, ATP-based hydrolysis has some differences, as we have also structurally demonstrated in the crystal structure of RT in complex with DNA and tetraphosphate excision product [49]. The above experiments provide strong evidence that K65R mutation confers hypersusceptibility to EFdA mainly through decreased excision.

## Discussion

Tenofovir is a major component of current antiviral therapies (http://aidsinfo.nih.gov/contentfiles/lvguidelines/adultandadolescentgl.pdf) and new HIV drugs are likely to be used in patients that have failed tenofovir-based treatment. Hence, the ability of novel HIV inhibitors to efficiently block tenofovir-resistant viruses is critical for their potential utility as HIV therapeutics. The clinical cut-off for tenofovir resistance is defined as a 2.1-fold reduction in virological response. It is associated with the presence of the tenofovir-resistance signature mutation K65R in the reverse transcriptase gene [50]. We report here that EFdA is highly potent against tenofovir-resistant K65R HIV, and inhibits this mutant 2.5-fold *more* efficiently than WT HIV. Given the fact that clinical resistance to tenofovir is considered a 2.1-fold decrease in susceptibility, we consider a 2-fold increase in susceptibility as significant hypersusceptibility. Understanding the mechanism by which HIV becomes resistant or more susceptible to EFdA could allow us to overcome drug resistance challenges and improve the current combination therapies. We have previously demonstrated that EFdA is highly efficient in suppressing viral replication of clinical isolates harboring signature mutations to other NRTIs and NNRTIs, including isolates containing 3TC/FTC resistance mutation M184V; TAMs or Q151M complex mutations that confer resistance to AZT, d4T, and abacavir; and nevirapine and efavirenz resistance mutations K103N and Y181C [45]. In addition, we have recently shown that EFdA is 3 logs more potent in SIV inhibition than tenofovir, AZT, and 3TC, and EFdA treatment decreases viral load in SIV-infected macaques by 3–4 logs within 1 week of SIV therapy and eventually to non-detectable levels [51]. The present study demonstrates that the K65R tenofovir-resistance RT mutation confers HIV hypersensitivity to EFdA compared to WT HIV. Other studies have shown that NRTI resistance mutations can confer enhanced susceptibility to other NRTIs. Specifically, the K65R and to a lesser extent the L74V RT mutations have been reported to suppress AZT resistance [43,52-55]. In addition, we have previously reported that K65R and L74V HIVs can be hypersusceptible to NRTIs with 4’-ethynyl substitutions [45,56]. The NNRTI-resistance mutation Y181C also increases susceptibility to AZT [57,58]. Moreover, the 3TC/FTC-resistance mutation M184V also increases HIV sensitivity to AZT by decreasing the excision efficiency of AZT-MP [22,53,59-61]. Finally, we have recently shown that the 172K polymorphism can enhance susceptibility to both NRTIs and NNRTIs [62].

**Figure 4:**
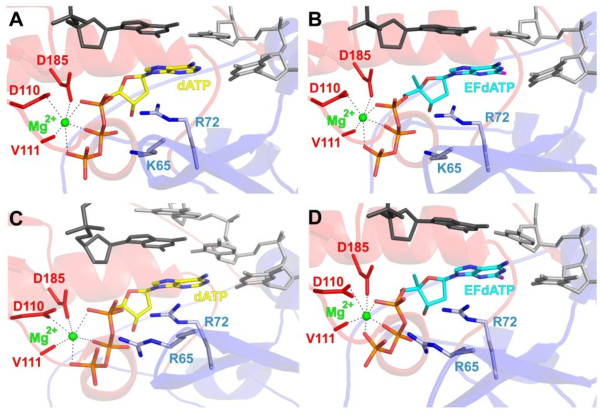
**Molecular models of dATP and EFdA-TP in the active sites of WT and K65R HIV RT.** dATP (yellow sticks, **A** and **C**) and EFdA-TP (cyan sticks, **B** and **D**) are shown at the active sites of WT HIV RT, (**A** and **B**) or K65R HIV RT (**C** and **D**). The fingers and palm subdomains are shown in blue and red cartoon, respectively. The primer and template strands are shown in dark gray and light gray sticks, respectively. Figures were made using PyMOL (The PyMOL Molecular Graphics System, Version 1.3 Schrödinger, LLC).

To determine whether the K65R RT mutation has the same effect at the enzyme level as well, we also carried out inhibitor susceptibility experiments with WT and K65R recombinant RT enzymes. Indeed, our enzymatic assays clearly showed that K65R RT is more susceptible to inhibition by EFdA-TP than WT RT. We thus focused on the biochemical mechanism of the enhanced EFdA susceptibility. We previously reported that EFdA is a TDRTI and inhibits primarily by blocking translocation after its incorporation at the 3’-end of the primer [45,46]. Hence, we investigated the effect of the K65R mutation on translocation using the site-specific Fe^2+^ footprinting assay. We found that K65R mutation has only a small effect on the translocation state of the EFdA-MP-terminated DNA·RT complex suggesting that the EFdA-MP-terminated primers stay at the nucleotide binding site (N site) of K65R RT as much as they do at the N site of WT RT. Since the EFdA resistance was not the result of changes in translocation efficiency, we hypothesized that K65R affects either the incorporation of the inhibitor itself, or its excision from EFdA-terminated primers. The effect on incorporation efficiency was assessed with single nucleotide incorporation experiments, whereas the effect on excision was measured in PPi- and ATP-dependent excision experiments under steady state conditions. Our results showed that the K65R mutation decreased the incorporation efficiencies of EFdA-TP and dATP to the same extent. Since pyrophosphorolysis is the reverse reaction of polymerization we hypothesized that it would also be slower in the presence of this mutation. This was confirmed by a PPi-based excision assay where we measured unblocking of EFdA-MP from the 3’-end of the primer. We found that K65R reduced excision and kept EFdA-MP-terminated primers blocked, explaining the hypersusceptibility that we have reported. In addition, when we used conditions that more closely mimic cell-based conditions, with ATP as the unblocking reagent and also all dNTPs present in the reaction to extend the unblocked primers, we also found that K65R reduced excision. Since the footprinting data did not show any significant difference in the translocation efficiency we can therefore conclude that the excision is not decreased because the EFdA-MP-terminated primers reside less at the excisable site. A decreased unblocking of EFdA-MP-terminated primers is not due to their inability to bind at the excisable N site of K65R RT. Instead, the molecular models in Figure 4 suggest that residues R65 and K65 interact differently with R72 and the phosphate moieties of EFdA-TP or dNTP, and thus may differentially affect the recognition of the pyrophosphate donor (ATP or PPi) and its nucleophilic attack on EFdA-terminated primers. Future crystallographic studies should provide more details on the molecular basis of excision-based EFdA resistance.

## Conclusion

We have provided virological and biochemical data demonstrating that the K65R RT mutation confers enhanced sensitivity to EFdA. We reported here that the mechanism of hypersensitivity is mainly through reduced excision of the chain terminating EFdA-MP. Our findings demonstrated that EFdA is a very potent NRTI and it could be used not only against WT HIV but also against tenofovir-resistant HIVs. The primary resistance mutation for EFdA is M184V and combination with tenofovir could be similar to the pair of mutations for 3TC/AZT combination. Unlike AZT and 3TC which are analogs of different deoxynucleosides, EFdA and tenofovir are both deoxyadenosine analogs and would theoretically compete to each other. However, they are activated/phosphorylated by different pathways [45]. Therefore, combination of EFdA with tenofovir could help suppress K65R resistance. This conclusion has significant potential therapeutic implications. Moreover, EFdA would be a good candidate in salvage therapies for patients that fail tenofovir-treatment due to K65R resistance.

## Methods

### Cells and viruses

TZM-bl cells (CCR5 transduced HeLa-CD4/LTR-β-gal and luciferase cells) were obtained from the AIDS Research and Reference Reagent Program, the National Institutes of Health (NIH). 293T and TZM-bl cells were maintained in Dulbecco’s Modified Eagle Medium supplemented with 10% fetal calf serum, 100 U/ml penicillin and 100 μg/ml streptomycin, and used for transfection and antiviral assays, respectively.

K65R RT mutation was introduced by site-directed mutagenesis as described previously [63,64]. Briefly, the desired mutations were introduced into the *Xma* I - *Nhe*I region (759 bp) of pTZNX1, which encodes nucleotides Gly-15 to Ala-267 of HIV-1 RT. After mutagenesis, the *Xma*I - *Nhe*I cassettes were inserted back into pNL101 and confirmed by sequencing. Viral stocks were obtained by transfection of each molecular clone into 293T cells using Fugene 6 (Roche, Mannheim, Germany), harvested and stored at −80°C until use.

### Cell-based drug susceptibility assays

Single-replication-cycle drug susceptibility assays were performed in triplicates using TZM-bl cells. TZM-bl cells were infected with diluted virus stock at 400,000 relative light units (RLU) in the presence of increasing concentrations of RTIs and cultured for 48 h. The luciferase marker gene expressions were measured using the Bright-Glo (Promega, WI). Susceptibility to NRTIs was calculated as the concentration that reduces RLU (infection) by 50% (50% effective concentration [EC_50_]). The data were obtained from the results of at least three independent experiments and the P values were determined using *t*-test statistical analysis.

### Enzymes and nucleic acids

HIV-1 RTs were expressed in JM-109 (Invitrogen) bacteria and purified by nickel affinity chromatography and monoQ anion exchange chromatography as previously described [46,65-69]. Oligonucleotides used in this study were chemically synthesized and purchased from Integrated DNA Technologies (Coralville, IA). Sequences of the DNA substrates are shown in Table 3. Deoxynucleotide triphosphates and dideoxynucleotide triphosphates were purchased from Fermentas (Glen Burnie, MD). EFdA was synthesized by Yamasa Corporation (Chiba, Japan) as described before [70]. Using EFdA as starting material the triphosphate form EFdA-TP was synthesized by TriLink BioTechnologies (San Diego, CA). Concentrations of nucleotides and EFdA-TP were calculated spectrophotometrically on the basis of absorption at 260 nm and their extinction coefficients. All nucleotides were treated with inorganic pyrophosphatase (Roche Diagnostics) as described previously [26] to remove traces of PPi contamination that might interfere with the rescue assay.

### Enzymatic drug susceptibility assays

#### Inhibition of HIV-1 RT-catalyzed DNA Synthesis by EFdA-TP

DNA template was annealed to 5’-Cy3 labeled DNA primer (3:1 molar ratio) (T_d31_/P_d18-P0_). To monitor primer extension, the DNA/DNA hybrid (20 nM) was incubated at 37°C with WT or K65R HIV-1 RT (20 nM) in a buffer containing 50 mM Tris (pH 7.8) and 50 mM NaCl (RT buffer). Subsequently, varying amounts of EFdA-TP were added and the reactions were initiated by the addition of 6 or 10 mM MgCl_2_ in a final volume of 20 μl. All dNTPs were present at a final concentration of 1 μM in the presence or absence of 3.5 mM ATP. The reactions were terminated after 50 minutes by adding equal volume of 100% formamide containing traces of bromophenol blue. The products were resolved on 15% polyacrylamide 7 M urea gels. In this and in subsequent assays the gels were scanned with a Typhoon FLA 9000 PhosphorImager (GE Healthcare, NJ). The bands corresponding to fully-extended product were quantified using Multi Gauge software. The results of at least four independent experiments were plotted as percent full extension using one site-competition nonlinear regression in GraphPad Prism 4 to determine the mean and standard deviation of the IC_50_ for EFdA-TP.

### Steady-state Kinetics

#### Single nucleotide incorporation of dATP and EFdA-TP by WT and K65R RTs

Steady-state kinetic parameters *K*_*m*_ and *k*_*cat*_ for incorporation of EFdA-TP or dATP were determined using single nucleotide incorporation in gel-based assays under saturating concentrations of T/P (10-fold excess over RT). Reactions were carried out in RT buffer, 6 mM MgCl_2_, 100 nM T_d26_/P_d18-P5_ or T_d31_/P_d18-P0_ or T_d31A_/P_d21_ (Table 3) and 10 nM WT or K65R HIV-1 RT in a final volume of 20 μl and stopped at indicated reaction times. The products were resolved and quantified as described above. *K*_*m*_ and *k*_*cat*_ were determined graphically using the Michaelis-Menten equation. Reactions were carried out in two to four independent experiments to determine the mean and standard deviation values.

### Site-specific Fe^2+^ footprinting assay

Site-specific Fe^2+^ footprints were monitored on 5'-Cy3-labeled DNA templates. 100 nM of 5’-Cy3-T_d43_/P_d30_ was incubated with 600 nM WT or K65R HIV-1 RT in a buffer containing 120 mM sodium cacodylate (pH 7), 20 mM NaCl, 6 mM MgCl_2_, and 1 μM EFdA-TP, to allow quantitative chain-termination. Prior to the treatment with Fe^2+^, complexes were pre-incubated for 7 min with increasing concentrations of the next incoming nucleotide (dTTP). The complexes were treated with ammonium iron sulfate (1 mM) as previously described [46,47]. This reaction relies on autoxidation of Fe^2+^[71] to create a local concentration of hydroxyl radicals, which cleave the DNA at the nucleotide closest to the Fe^2+^ specifically bound to the RNase H active site. These experiments were performed at least twice.

### ATP- and PPi-dependent excision and rescue of T/P_EFdA-MP_

#### ATP-dependent rescue of T/P_EFdA-MP_

Template/primer with EFdA-MP at the 3’ primer terminus (T/P_EFdA-MP_) was prepared by incubating 500 nM T_d31_/P_d18-P0_ with 1 μM HIV-1 RT in RT buffer and 6 mM MgCl_2_. EFdA-TP was added into the reaction and the mixture was incubated at 37°C for 1 h. After incorporation of EFdA-TP, the T/P_EFdA-MP_ was purified using the QIAquick nucleotide removal kit (Qiagen, Valencia, CA). Under these conditions, the extension of T/P to T/P_EFdA-MP_ was complete. 20 nM of purified T_d31_/P_d18-P0-EFdA-MP_ was incubated with 60 nM WT or K65R HIV-1 RT in the presence of 3.5 mM ATP, 100 μM dATP, 0.5 μM dTTP, and 10 μM ddGTP in RT buffer and 10 mM MgCl_2_. Aliquots of the reaction were stopped at different time points (0–90 min). The data from at least four independent experiments were analyzed using GraphPad Prism 4.

#### PPi-dependent rescue of T/P_EFdA-MP_

20 nM of purified T_d31_/P_d18-P0-EFdA-MP_ was incubated at 37°C with 60 nM WT or K65R HIV-1 RT in the presence of 150 μM PPi, 100 μM dATP, 0.5 μM dTTP, and 10 μM ddGTP in RT buffer and 6 mM MgCl_2_. Aliquots of the reaction were stopped at different times (0–40 min). The data from at least two independent experiments were plotted using GraphPad Prism 4.

### Molecular modeling

Molecular models of dATP and EFdA-TP in the active site of WT HIV RT were made using PDB ID 1 T05 [72] as a starting model (WT HIV RT in complex with tenofovir diphosphate). A molecular model of EFdA-TP in the active site of K65R HIV RT was made using PDB ID 3JYT [44] as a starting model (K65R HIV RT in complex with dATP). The sketch module of SYBYL (Version 7.3.5, Tripos International, St. Louis, MO) was used to make dATP and EFdA-TP molecules. dATP and EFdA-TP were each superposed to tenofovir diphosphate in the WT complex, after which the tenofovir diphosphate was removed. Gasteiger-Huckel charges were calculated and molecular minimization of the WT-dATP and WT-EFdA-TP were performed in SYBYL using the Powell method. SYBYL was also used to add the 2-fluoro and 4’-ethynyl groups to dATP in the K65R complex. Gasteiger-Huckel charges were then calculated and molecular minimization was performed as for the WT complexes.

## Abbreviations

HIV: Human immunodeficiency virus; RT: Reverse transcriptase; NRTI: Nucleoside reverse transcriptase inhibitor; TDRTI: Translocation-defective RT inhibitor; EFdA: 4'-ethynyl-2-fluoro-2'-deoxyadenosine; MP: Monophosphate; TP: Triphosphate; TDF: Tenofovir disoproxil fumarate; T/P: Template/primer; T/PEFdA-MP: Template/primer possessing EFdA-MP at the 3'-primer terminus (or T/P chain terminated by EFdA-MP).

## Competing interests

Hiroaki Mitsuya and Eiichi Kodama are inventors of EFdA.

## Authors’ contributions

EM designed the biochemical experiments. EM and EMR carried out the biochemical experiments. MDL, ADH, and KS assisted in some of the biochemical experiments. AH designed and carried out the cell-based assays. KAK and SGS performed the molecular modeling studies. YTO and JCJ participated in the initial biochemical studies. BM participated in the design of biochemical experiments and interpretation of data. ENK, HM, and MAP helped with preliminary virological data. MAP helped in the data interpretation. EM drafted the manuscript. SGS conceived and coordinated the study and drafted the manuscript. All authors read and approved the final manuscript.

## Supplementary Material

Additional file 1: Figure S1 Effect of K65R mutation on the formation of RT:T/P_EFdA-MP_ complex. Purified T/P_EFdA-MP_ (25 nM) was incubated at room temperature for 10 min with different concentrations of WT or K65R RTs in RT buffer and 6 mM MgCl_2_. RT was used at different concentrations to obtain RT:DNA ratios that ranged from 0 to 10. Four μl of 20% sucrose was added to each mixture in a final volume of 24 μl. The complexes were subsequently resolved on a native 6% polyacrylamide Tris borate gel and visualized as described in Methods. Click here for file
