# Supplementary material for: Hypersusceptibility mechanism of Tenofovir-resistant HIV to EFdA
Source: Retrovirology. 2013 Jun 24;10:65. doi: 10.1186/1742-4690-10-65 (PMC3695782; doi:10.1186/1742-4690-10-65)
Supplement: Additional file 1: Figure S1 — Effect of K65R mutation on the formation of RT:T/PEFdA-MP complex. Purified T/PEFdA-MP (25 nM) was incubated at room temperature for 10 min with different concentrations of WT or K65R RTs in RT buffer and 6 mM MgCl2. RT was used at different concentrations to obtain RT:DNA ratios that ranged from 0 to 10. Four μl of 20% sucrose was added to each mixture in a final volume of 24 μl. The complexes were subsequently resolved on a native 6% polyacrylamide Tris borate gel and visualized as described in Methods. [file 1742-4690-10-65-S1.pptx]

## Slide 1
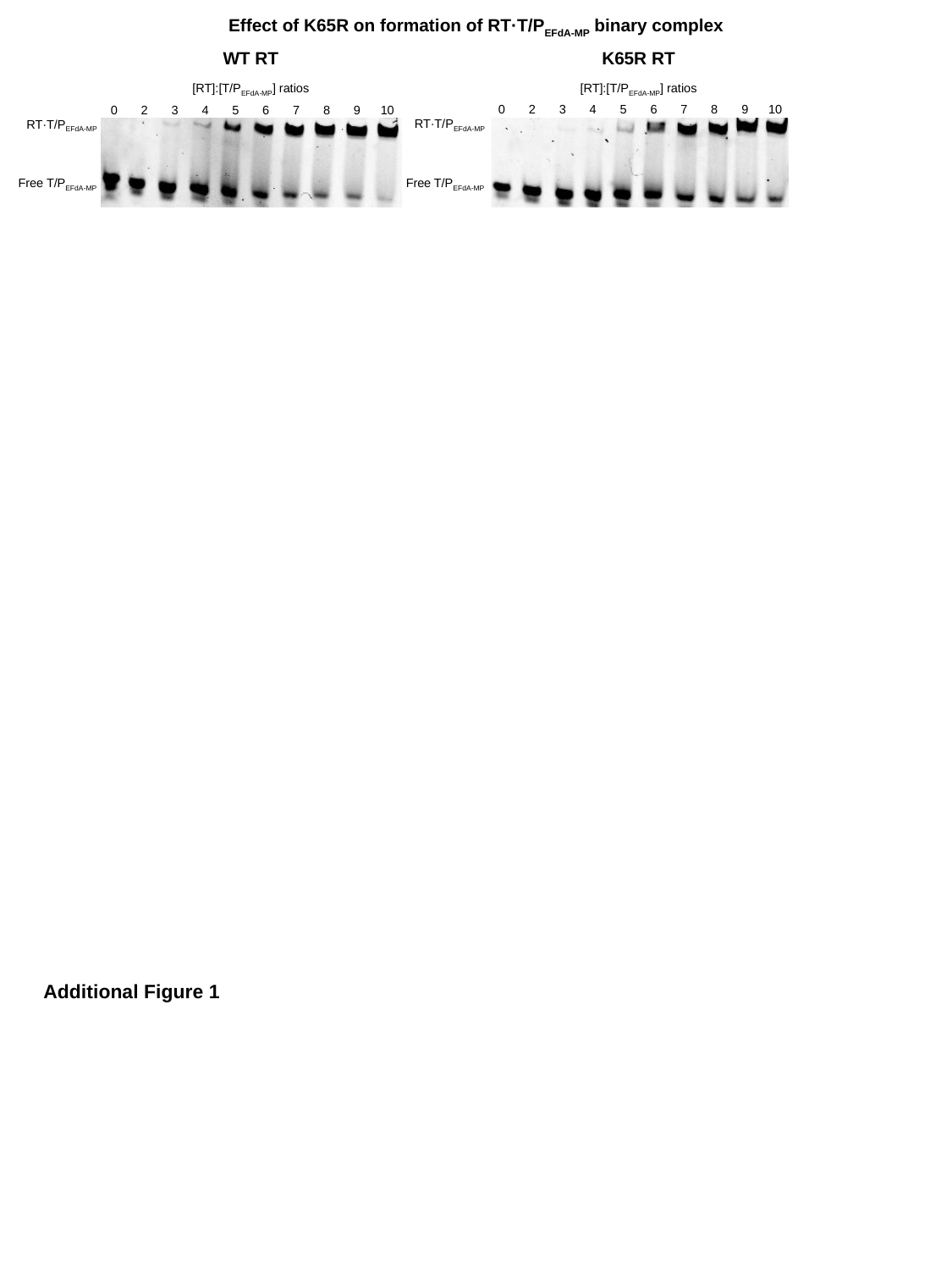

Effect of K65R on formation of RT·T/PEFdA-MP binary complex
K65R RT
WT RT
[RT]:[T/PEFdA-MP] ratios
[RT]:[T/PEFdA-MP] ratios
0
2
3
4
5
6
7
8
9
10
0
2
3
4
5
6
7
8
9
10
RT·T/PEFdA-MP
RT·T/PEFdA-MP
Free T/PEFdA-MP
Free T/PEFdA-MP
Additional Figure 1
